# Cilia-Localized Counterregulatory Signals as Drivers of Renal Cystogenesis

**DOI:** 10.3389/fmolb.2022.936070

**Published:** 2022-06-23

**Authors:** Rebecca V. Walker, Anthony Maranto, Vivek Reddy Palicharla, Sun-Hee Hwang, Saikat Mukhopadhyay, Feng Qian

**Affiliations:** ^1^ Division of Nephrology, Department of Medicine, University of Maryland School of Medicine, Baltimore, MD, United States; ^2^ Department of Cell Biology, UT Southwestern Medical Center, Dallas, TX, United States

**Keywords:** polycystic kidney disease, cystogenesis, primary cilia, polycystin 1 and 2, tubby-like protein 3, cilia-dependent cyst activation, cilia-localized cyst inhibition, intraflagellar transport

## Abstract

Primary cilia play counterregulatory roles in cystogenesis—they inhibit cyst formation in the normal renal tubule but promote cyst growth when the function of polycystins is impaired. Key upstream cilia-specific signals and components involved in driving cystogenesis have remained elusive. Recent studies of the tubby family protein, Tubby-like protein 3 (TULP3), have provided new insights into the cilia-localized mechanisms that determine cyst growth. TULP3 is a key adapter of the intraflagellar transport complex A (IFT-A) in the trafficking of multiple proteins specifically into the ciliary membrane. Loss of TULP3 results in the selective exclusion of its cargoes from cilia without affecting their extraciliary pools and without disrupting cilia or IFT-A complex integrity. Epistasis analyses have indicated that TULP3 inhibits cystogenesis independently of the polycystins during kidney development but promotes cystogenesis in adults when polycystins are lacking. In this review, we discuss the current model of the cilia-dependent cyst activation (CDCA) mechanism in autosomal dominant polycystic kidney disease (ADPKD) and consider the possible roles of ciliary and extraciliary polycystins in regulating CDCA. We then describe the limitations of this model in not fully accounting for how cilia single knockouts cause significant cystic changes either in the presence or absence of polycystins. Based on available data from TULP3/IFT-A-mediated differential regulation of cystogenesis in kidneys with deletion of polycystins either during development or in adulthood, we hypothesize the existence of cilia-localized components of CDCA (cCDCA) and cilia-localized cyst inhibition (CLCI) signals. We develop the criteria for cCDCA/CLCI signals and discuss potential TULP3 cargoes as possible cilia-localized components that determine cystogenesis in kidneys during development and in adult mice.

## Introduction

Autosomal dominant polycystic kidney disease (ADPKD) is characterized by the formation of numerous fluid-filled cysts (Igarashi and Somlo, 2002; Harris and Torres, 2009) from all tubule segment origins. The cysts destroy normal parenchyma and cause kidney failure in more than half of the patients by the age of 60 (Gabow, 1993). ADPKD is caused primarily by mutations in *PKD1* or *PKD2*, which encodes polycystin-1 (PC1) or polycystin-2 (PC2), respectively (polycystins or PCs collectively) (The European Polycystic Kidney Disease Consortium, 1994; Mochizuki et al., 1996). PC1 is a 4302 aa 11-transmembrane (TM) protein (Hughes et al., 1995) with a GPCR autoproteolysis-inducing (GAIN) domain located upstream of the first TM domain (Ponting et al., 1999; Arac et al., 2012). PC1 undergoes autoproteolytic cleavage at a G protein-coupled receptor (GPCR) proteolysis site (GPS) within the GAIN domain—a key post-translational modification of the protein (Qian et al., 2002; Yu et al., 2007; Chapin et al., 2010; Cai et al., 2014; Kim et al., 2014; Kurbegovic et al., 2014; Gainullin et al., 2015; Padovano et al., 2020). PC2 belongs to the transient receptor potential (TRP) channel superfamily of proteins (Koulen et al., 2002) and can form a homotetramer that functions as a non-selective cation channel (Shen et al., 2016; Grieben et al., 2017). PC1 and PC2 form a stable complex with a 1:3 stoichiometry (Yu et al., 2009; Zhu et al., 2011; Su et al., 2018). Recent studies have shown that PC1/PC2 complex has an ion channel function with properties that are distinct from that of the homomeric PC2 channel (Wang et al., 2019; Ha et al., 2020). Moreover, the PC1 subunit directly contributes to the channel pore and thereby affects its ion channel function (Wang et al., 2019). Polycystins transduce intracellular calcium signals in response to extracellular stimuli (Nauli et al., 2003; Delmas, 2004; Nauli and Zhou, 2004) by unknown mechanisms. Multiple downstream cellular pathways, such as extracellular regulated kinase (Shibazaki et al., 2008; Ma et al., 2013), mTOR (Shillingford et al., 2006; Boletta, 2009; Distefano et al., 2009), cAMP (Torres et al., 2004; Wang et al., 2018), WNT (Kim et al., 2016), Ca^2+^ (Koulen et al., 2002; Nauli et al., 2003; Cantiello, 2004; Anyatonwu et al., 2007; Kim et al., 2016) and G-protein signaling (Parnell et al., 1998; Hama and Park, 2016; Parnell et al., 2018; Zhang et al., 2018) are dysregulated in polycystic kidneys with mutated polycystins. How these downstream pathways are mechanistically linked to cystogenesis caused by a lack of polycystins remains largely unknown. Although current therapies target many of these pathways, they have achieved limited effects and showed significant side effects that restrict their usage (Torres et al., 2010; Torres and Harris, 2014; Torres et al., 2017; Torres et al., 2020). Many therapies are targeted toward preventing the progression of the disease rather than halting disease initiation. Indeed, distinguishing the changes that arise from initiation of, and are proximate to, cystogenesis from those that are secondary to the progression of cysts has been challenging. Key upstream signals that are regulated by polycystins and determine kidney cystogenesis in ADPKD remain unknown, preventing the development of more effective therapies.

## Complex Role of the Primary Cilium in Cystogenesis

An important clue into the role of the polycystins in tubular homeostasis came from the findings that both proteins localize to the primary cilium (Pazour et al., 2002; Yoder et al., 2002; Nauli et al., 2003; Nauli and Zhou, 2004). The primary cilium is a small hair-like projection from the apical surface of almost all cell types (Rosenbaum and Witman, 2002; Wheatley, 2005). Primary cilia function as sensory antennae in most vertebrate cells playing fundamental roles in cell cycle control, cellular differentiation, and polarity (Goetz and Anderson, 2010; Anvarian et al., 2019). In the kidney, primary cilia jut out into the tubular lumen and are thought to sense urine flow-induced shear stress or ion composition (Nauli et al., 2003; Praetorius et al., 2003).

The ciliary localization of polycystins has led ADPKD to be categorized as a ciliopathy, a genetic disease caused by defective cilium-resident proteins and ciliary dysfunction. In fact, kidney cyst formation during development appears to be common pathogenesis for many other ciliopathies. These results suggest that cilia regulate tubular homeostasis and that ciliary dysfunction is a key upstream pathogenic step leading to tubular dilatation and cystogenesis (Pazour, 2004; Nauli et al., 2006). It has been suggested that ciliary polycystins may sense mechanical stimuli by mediating calcium entry into cilia, and loss of the mechanically induced cilia-initiated calcium signaling may underlie cystogenesis (Nauli et al., 2003). This view has recently been challenged by the Clapham laboratory who demonstrated that primary cilia are not calcium-responsive mechanosensors (Delling et al., 2016).

**FIGURE 1:**
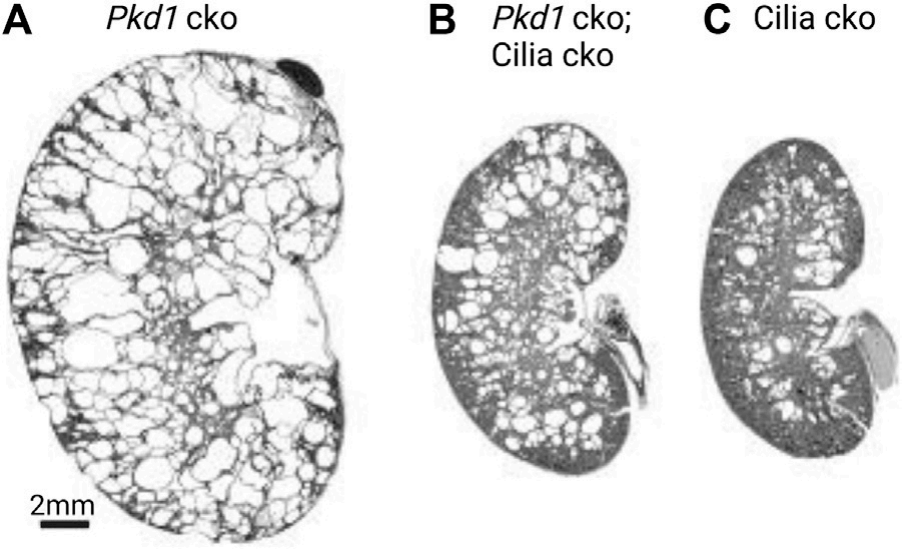
Complex roles of cilia in cystogenesis. Cysts from PC1 loss are severe and only partially suppressed from cilia loss. Images adapted from (Ma et al., 2013) with permission. Postnatal day 24 (P24) kidneys from **(A)** *Pkd1* cko (*Pkhd1-cre;Pkd1* ^*fl/fl*^)*,* **(B)** *Pkd1* cko*;* cilia cko (*Pkhd1-cre;Kif3a* ^*fl/-*^ *;pkd1* ^*fl/fl*^)*,* and **(C)** cilia cko (*Pkhd1-cre;Kif3a* ^*fl/-*^) mice. Abbreviations: cko, conditional knockout.

Genetic studies have shown a complex role of primary cilia in cystogenesis. Loss of cilia, by deletion of the intraflagellar transport complex-B proteins (Davenport et al., 2007; Jonassen et al., 2008) or kinesin-II (Lin et al., 2003), causes mild fibrocystic disease with slow cyst growth in the kidney. This finding suggested that cilia inhibit cyst formation in normal renal tubules. By comparison, loss of the polycystins caused significantly more severe cystogenesis marked by early and rapid cyst growth while keeping intact ciliary structure (Lu et al., 1997; Boulter et al., 2001; Piontek et al., 2004), for example see Figure 1A. In a pioneering study, the Somlo laboratory has shown that concomitant loss of polycystins and cilia results in an intermediate phenotype (Figure 1B), closer to that of loss of cilia alone (Figure 1C) (Ma et al., 2013). Therefore, polycystins play an important role in cilia signaling to suppress tubular dilation and cystogenesis. Furthermore, the extent of the suppression was directly related to the length of time between the initial loss of the polycystins and the subsequent involution of cilia—the disease worsened when this time interval was lengthened (Ma et al., 2013). The suppression of cyst growth by the loss of cilia was found to occur in all segments of the renal tubules following both early and late *Pkd1* or *Pkd2* gene deletion. The genetic epistasis data indicate that primary cilia play dual and opposing roles in cyst development dependent on the presence of polycystins: they inhibit cyst formation in the normal renal tubule but promote cyst growth when polycystins are lacking.

## PC1/PC2 Repressed Cilia-Dependent Cyst Activation Signal

**FIGURE 2:**
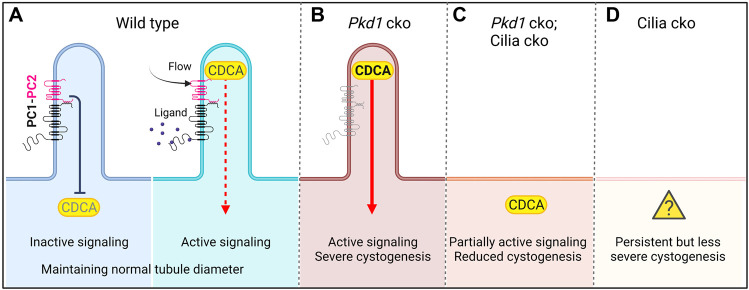
Cilia-dependent cyst activation (CDCA) model for cystogenesis in ADPKD (adapted from Ma, 2021). **(A)** The CDCA in the wild-type cell is suppressed by polycystins and is activated through an unknown mechanism, possibly ligand binding to polycystins or flow bending the cilium. **(B)** In *Pkd1* cko, CDCA is derepressed and constitutively activated leading to severe cystogenesis. **(C)** If *Pkd1* and cilia are co-ablated, the CDCA cannot be upregulated. The cystogenic signal is thus diminished compared with single *Pkd1* cko, resulting in reduced cystogenesis. In this model, the CDCA signal must retain a partial “leaky” function in the cytoplasm to explain how *Pkd1*-cilia double mutants display significant cystic changes. **(D)** In cilia single mutants, there is persistent mild cystogenesis. Here the CDCA must have a leaky function despite the presence of intact polycystins and lack of cilia (**“?”**). Created in BioRender.

Based on the above-mentioned genetic epistasis data from *Pkd1/Pkd2* and cilia conditional knockout (cko) mice, the Somlo laboratory has proposed an unidentified cilia-dependent cyst activation (CDCA) signal(s) (Ma et al., 2013; Ma et al., 2017). The CDCA signal is dependent on intact cilia for activity and is normally inhibited by the functioning of polycystins (Figure 2A). The function of polycystins in suppressing CDCA might be part of a normal physiological or homeostatic cilia-dependent signaling pathway promoting functional tubule adaptation to either chemical or mechanical signals (Ma et al., 2013). Loss of polycystins in the presence of intact cilia—the condition for cyst initiation in ADPKD—leads to upregulation of CDCA, perhaps *via* ciliary translocation, and constitutive activation (Figure 2B). This leads to uncontrolled lumen diameter expansion and cyst growth (Ma et al., 2013; Ma et al., 2017; Ma, 2021). Ciliary localization of CDCA is required for full activation in *Pkd1* cko; the CDCA signal is impeded when cilia are co-ablated, thus resulting in a reduction of the cystic burden in the *Pkd1*-cilia double mutants (Figure 2C).

The molecular nature of CDCA components and effectors are unknown. The CDCA mechanism of cyst growth is active in all tubular segments following both early and late ADPKD gene inactivation (Ma et al., 2013). The CDCA signal(s) is likely distinct from MAPK/ERK (Shibazaki et al., 2008; Distefano et al., 2009), mTOR (Pema et al., 2016), cAMP (Torres and Harris, 2014; Bergmann et al., 2018), and phosphorylated cAMP Response Element-Binding Protein (pCREB) signaling (Kakade et al., 2016) at a cellular level (Ma et al., 2013), which are altered in cystic kidneys. These pathways are unlikely to be central to the CDCA mechanism because they show restricted nephron segment specificities and later activation (Ma et al., 2013). These pathways may be activated only in certain cell types or conditions to actively promote cyst growth, making them unlikely to be universally applicable targets for reducing cyst growth. A recent study has shown that signaling through the Hedgehog pathway is not required for cystic phenotype caused by loss of function of *Pkd1* (Ma et al., 2019). In fact, neither activation nor repression of the Hedgehog signaling components (*Smo*, *Gli2*, and *Gli3*) influenced the progression of polycystic kidney disease in mouse *Pkd1* models of developmental or adult-onset of ADPKD. These results suggested that the Hedgehog signaling pathway does not contribute to the CDCA or other ciliary signals that drive renal cystogenesis.

## Roles of Ciliary and Extraciliary Polycystins in Regulating Cilia-Dependent Signaling

PC1 and PC2 localize to multiple subcellular compartments in the cell body in addition to cilia where they can exert their various functions (Boletta and Germino, 2003; Kottgen and Walz, 2005; Chapin and Caplan, 2010). This raises the question as to which of the polycystins pools may be involved in regulating the cilia-dependent signaling. Ciliary polycystins may inhibit the CDCA within the cilia, but direct evidence is lacking. Recent data suggest a critical role of the ciliary polycystins in this role. Walker et al. (2019) have analyzed Pkd2lrm4 mutant model, a missense (E442G) mutant variant that encodes a channel-functional (Yoshiba et al., 2012) but non-cilia localizing (Grimes et al., 2016; Walker et al., 2019) form of PC2. The mutant mice developed a *Pkd2* null-like phenotype characterized by embryonic kidney cysts (Walker et al., 2019). This finding suggests that ciliary exclusion of PC2 is sufficient to cause kidney cystogenesis in a mouse model of ADPKD. Similarly, PC2^W414G^, a human pathogenic variant, retains its channel activity but fails to traffic to the cilia (Cai et al., 2014). Cai et al. (2014) examined the ciliary localization of a cohort of human missense pathogenic variants of PC1 and PC2 and found that ∼70% of them exhibit defects in ciliary trafficking. These data suggest that ciliary polycystins may be necessary to prevent kidney cyst formation and functional polycystins remaining in the cell body is not sufficient to counter the cystogenic signal from the cilium. The molecular mechanism by which the ciliary pool of polycystins inhibits the CDCA is unknown.

A critical role of the ciliary PC1 is further suggested by the finding of a GPS cleavage resistant mouse PC1 mutant with an amino acid substitution L3040H within the GAIN/GPS domain (Cai et al., 2014). This mutant did not reach cilia and its expression by a BAC transgene could not rescue embryonic cystogenesis and lethality in mouse *Pkd1* mutant background. However, the PC1 mutant (L3040H) expressed in the cell body remains completely Endoglycosidase H (Endo H) sensitive implying that this mutant is retained in the ER and defective in the trafficking (Cai et al., 2014). It is thus unclear whether loss-of-function observed for the PC1-L3040H mutant may be a secondary consequence of the unfolding of the GAIN domain or global structural disruption, making it difficult to conclude a role of the extraciliary pool of PC1. Interestingly, studies of the hypomorphic *Pkd1* knock-in model, *Pkd1* ^*V/V*^, provided evidence for a role of the extraciliary pools of polycystins in regulating the cilia-dependent signals (Yu et al., 2007). The *Pkd1* ^*V/V*^ mouse contains a single amino acid substitution T3041V at the GAIN/GPS domain, which blocks autoproteolytic cleavage of PC1 (Qian et al., 2002; Yu et al., 2007; Trudel et al., 2016). The resulting non-cleavable PC1^V^ mutant is excluded from cilia without disrupting the cilia (Kim et al., 2014). The *Pkd1* ^*V/V*^ mice escaped renal cystogenesis and lethality during embryonic stages that are seen in *Pkd1* null models but started cystic dilation in distal nephron segments and collecting ducts at birth, culminating in death at ∼3 weeks of age (Yu et al., 2007). The lack of ciliary access of this mutant PC1 form supports a mechanism that a CDCA signal is generated in intact cilia that are devoid of polycystins. Molecular analyses of the uncleavable PC1^V^ mutant protein showed that this mutant acquires Endo H resistance and thus can exit the ER and is functional for intracellular trafficking (Kim et al., 2014; Kurbegovic et al., 2014; Trudel et al., 2016). We recently showed that this extraciliary PC1 can form a functional ion channel complex with PC2 in *Xenopus* oocytes (Wang et al., 2019). The delayed and restricted cystogenesis in the *Pkd1* ^*V/V*^ model thus suggested that the extra-ciliary PC1 likely suppresses CDCA or its effector pathway(s), albeit less effectively than the wild-type protein. It remains to be seen whether the anti-cystogenic role of PC1^V^ in the cell body is mediated through its function in the mitochondria (Padovano et al., 2017) or *via* interaction with the cell matrix (Lee et al., 2015) or by the ion channel function of the polycystin complex at extraciliary membranes (Wang et al., 2019; Ha et al., 2020; Vien et al., 2020).

## Cyst Formation in Cilia Mutants: Cilia-Regulated Signals That Suppress Cystogenesis Independent of Polycystins

The concomitant ablation of cilia significantly suppressed rapid cyst growth in *Pkd1* cko compared with *Pkd1* cko single mutants. However, instead of completely resolving renal cyst development, this suppression was only partial, resulting in an intermediate phenotype of cyst growth, closer to that of loss of cilia alone (Ma et al., 2013). This was described by Ma et al. (2013), which shows that *Pkd1*-cilia double cko produced phenotypes more severe than cilia cko but much less severe than *Pkd1* cko when measured by the kidney to body weight ratio, cystic index, and serum urea nitrogen. The intermediate phenotype was consistent for both *Pkd1* and *Pkd2* mutants when in combination with a cilia cko, suggesting that this is a consistent phenomenon for the interaction between polycystins and cilia. This observation highlights several gaps in the CDCA model as it stands. First, cilia single knockouts cause significant cystic changes in the presence of polycystins, which should otherwise inhibit the CDCA to prevent cystogenesis in normal tubules (Fig. 1C). Without cilia, the cilia-dependent signal should be abolished and the cyst inhibiting role of polycystins should prevail. Second, cyst growth continues to persist in *Pkd1*-cilia double mutants (Fig. 1B); however, loss of cilia should prevent CDCA from initiating cystogenesis if the cyst activation signal solely originates in the cilium. Moreover, neither concomitant reduction in dose (by half) of PC1 nor transgenic overexpression of PC1 were found to have an impact on the cystic burden in cilia mutants (Ma et al., 2013). These data indicate that cysts from cilia single knockouts arise independently of PC1.

The above observations have additional implications. First, and according to the model (Ma et al., 2017; Ma, 2021) (Figure 2), the CDCA signal has to retain a partial “leaky” function or quiescent state in the cytoplasm to explain how *Pkd1*-cilia double mutants display significant cystic changes. Second, in cilia single mutants the CDCA has to be activated in absence of cilia and in the presence of intact polycystins to develop cysts (Figure 2D, **“?”**). An alternative parsimonious interpretation would be that persistent cyst growth in *Pkd1*-cilia double mutants arises from additional ciliary roles independent of polycystins. Cilia-defective mutants are likely to cause fibrocystic kidney disease phenotype by mechanisms (Jonassen et al., 2008; Choi et al., 2011) that are likely divergent from loss of polycystins (Shibazaki et al., 2008). The cilia-regulated signal(s) could normally suppress cystogenesis, parallel to the CDCA. Such signal(s) would require localization to cilia for full activity and cannot be generated if cilia are ablated as in cilia-single and *Pkd1*-cilia double mutants to suppress cystogenesis. We annotate this polycystin-independent ciliary component as cilia-localized cyst inhibition (CLCI) signal(s).

Overall, these considerations suggest a more complex cilia-regulated mechanism in cyst growth involving a combination of positive and negative regulatory signals in ADPKD. These counterregulatory signals generated within the cilium are in a finely tuned balance to facilitate functional tubule adaptation to physiological inputs in normal kidneys. Dysregulation of these ciliary signals may underlie cystic kidney diseases.

## Cilia-Dependent Signaling Within Cilia: Challenges of Assessment

Several difficulties have limited our ability to dissect the crucial ciliary signals. First, ciliary signals cannot be identified in experimental models that lack cilia. Ablation of cilia, an important cellular compartment, disrupts a great number of cellular pathways, including cell cycle regulation and cell polarity (Anvarian et al., 2019). The crude disruption of cilia, therefore, obscures our ability to determine the nature of the ciliary signals that are pertinent to cystogenesis. Identification of these ciliary signals will necessitate approaches that retain intact cilia. Second, uncoupling ciliary signals causing tissue phenotypes, such as cystogenesis, from the downstream pathways affected is difficult (Mukhopadhyay et al., 2017). Third, the small size of the cilium with respect to the cell (Delling et al., 2013) makes ciliary perturbations difficult to detect. Fourth, ciliary proteins, including PC1 and PC2 (Pisitkun et al., 2004; Hogan et al., 2009; Wang et al., 2015; Hardy and Tsiokas, 2020; Hu and Harris, 2020; Lea et al., 2020) have additional extraciliary roles necessitating approaches that selectively target ciliary pools of signaling proteins.

The components of the CDCA signal may be present in cilia and cytoplasm (Ma et al., 2017; Ma, 2021), but their relative contributions remain unclear. A recent transcriptomic study using *Pkd1* single and *Pkd1-*cilia double mutant kidneys has identified non-ciliary cyclin-dependent kinase 1 as a driver of cyst cell proliferation from *Pkd1* inactivation but did not find changes in ciliary drivers in cystogenesis (Zhang et al., 2021). The lack of detection of the ciliary drivers in the study may reflect heterogenous cystic mechanisms between polycystin and cilia loss. Alternatively, changes in ciliary signaling or trafficking of ciliary components may be too small to be detected at the global transcriptional level and are unlikely to be detected by the whole kidney analyses.

## Tubby-Like Protein 3 is a key Adapter of the IFT-A Complex in Trafficking Multiple Proteins to the Ciliary Membrane

**FIGURE 3:**
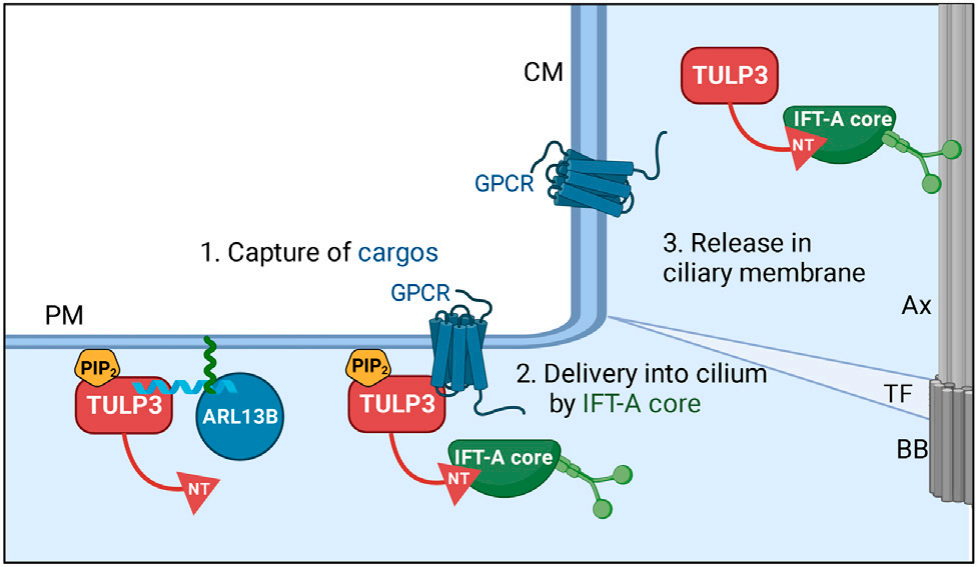
Model for TULP3 and IFT-A—mediated trafficking of cargoes into cilia. TULP3 tubby domain is anchored to the plasma membrane by PI(4,5)P_2._ The tubby domain captures short CLS (cilia localization signal) peptide regions in diverse cargoes. The N-terminus (NT) of TULP3 binds to the IFT-A core subunits (IFT140, IFT122, IFT144) and recruits the tubby domain-bound cargoes to cilia. After reaching cilia, the lack of PI(4,5)P_2_ in the ciliary membrane could dislodge the cargoes from the tubby domain. The cargoes are very diverse and include transmembrane proteins and membrane-associated proteins such as ARL13B. The N-terminus amphipathic helix in ARL13B that binds the tubby domain in TULP3 is shown in blue. ARL13B is anchored to the membrane by palmitoylation (green) inside the helix. Abbreviations: Ax, Axoneme; TF, transition fiber; BB, Basal body; CM, ciliary membrane; PM, plasma membrane. Created in BioRender.

The tubby family member, Tubby-like protein 3 (TULP3) is a key adapter of the intraflagellar transport complex A (IFT-A) in the trafficking of multiple proteins specifically into the ciliary membrane. The IFT-A holo-complex is generally considered to be regulating the retrograde trafficking of cargoes including IFT-B complex in the cilia (Piperno et al., 1998; Iomini et al., 2001; Iomini et al., 2009). TULP3 interacts with the IFT-A core (consisting of IFT140/122/144 subunits) (Mukhopadhyay et al., 2010; Behal et al., 2012) through its N-terminus to enable trafficking of itself and TULP3 bound cargoes into cilia (Mukhopadhyay et al., 2010; Badgandi et al., 2017). Thus, the IFT-A core complex has an additional function in the pre-ciliary trafficking of TULP3 and cargoes, in addition to its established role in retrograde trafficking in cilia. TULP3 mediates ciliary trafficking by a 3-step mechanism: 1) capture of membrane cargo by the tubby domain in a PI(4,5)P_2_-dependent manner, 2) ciliary delivery by IFT-A core-binding to TULP3 N-terminus, and 3) release into PIP_2_-deficient ciliary membrane (Figure 3) (Badgandi et al., 2017). Loss of TULP3 results in the selective exclusion of its cargoes from cilia without affecting their extraciliary pools and without disrupting cilia or IFT-A complex integrity. Therefore, studying TULP3 provides a unique opportunity to investigate potential ciliary components that regulate cystogenesis from intact cilia.

The IFT-A core and peripheral subunit mutants also affect the ciliary localization of multiple TULP3 cargoes (Fu et al., 2016; Takahara et al., 2018; Picariello et al., 2019; Kobayashi et al., 2021; Quidwai et al., 2021) indicating that IFT-A is required for their ciliary trafficking. Substitution of Ift140 subunit in *Chlamydomonas* with a WD-40 repeat deleted Ift140 fragment restores flagella partially, as opposed to lack in *Ift140* knockout. Instead, trafficking of multiple lipidated proteins, including ARL13 and other farnesylated and myristoylated proteins, are affected. Thus IFT-A could have an evolutionary function in the pre-ciliary trafficking of cargoes to cilia.

Recent genetic studies of *Tulp3* provided compelling evidence for cilia-localized signals in determining cyst growth. *Tulp3* depletion has been shown to be deleterious but also protective in developmental (Hwang et al., 2019) and adult-onset ADPKD models in mice (Legue and Liem, 2019). These results are described in detail below and they provide a unique approach to test potential TULP3 cargoes as possible components of the cilia-localized signals that determine cystogenesis.

## TULP3/IFT-A Ciliary Cargoes as Cilia-Localized Cyst Inhibition signal(s) During Development

**FIGURE 4:**
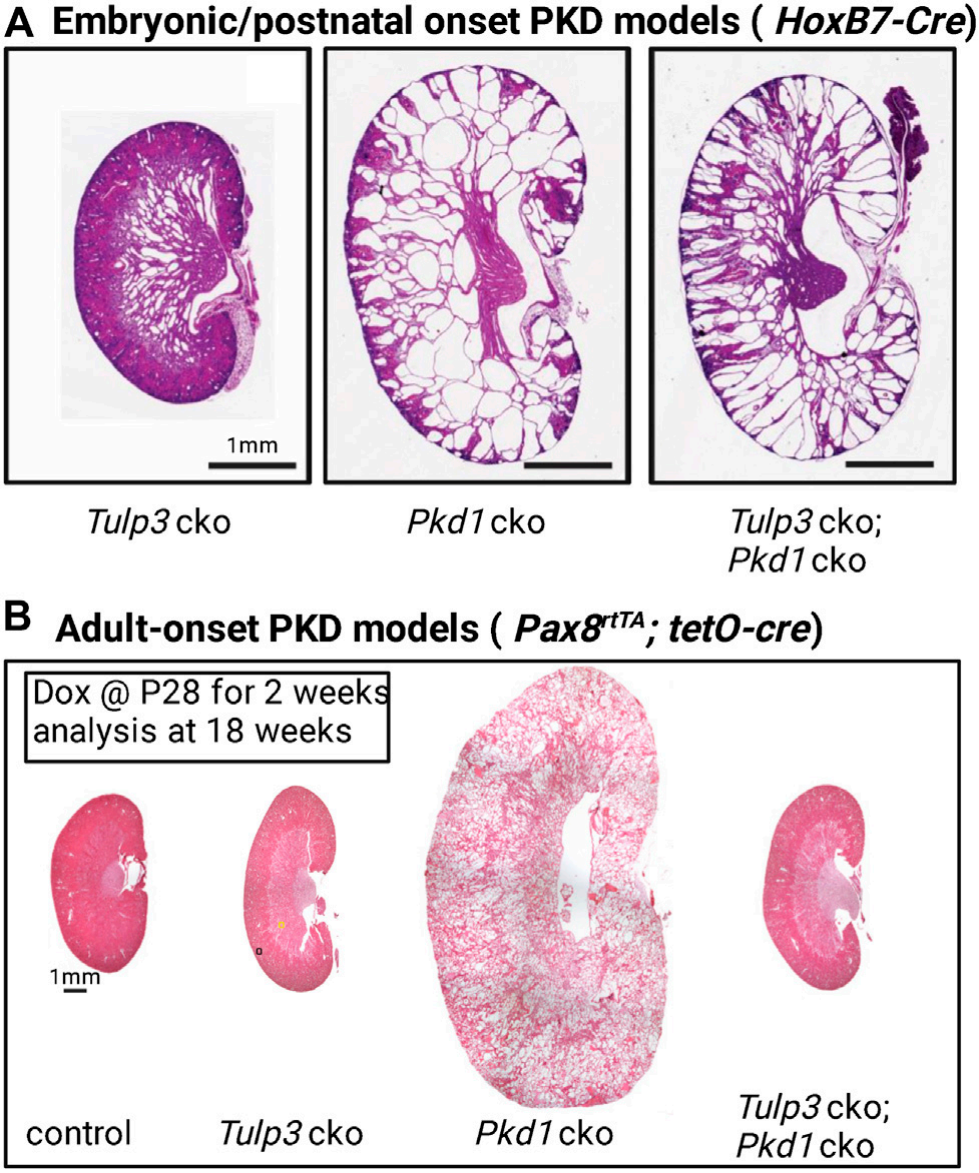
*Tulp3* and *Pkd1* double knockout models of PKD. **(A)** Kidneys from postnatal day 5 (P5). *Tulp3* cko (*HoxB7-cre; Tulp3* ^*fl/fl*^), *Pkd1* cko (*HoxB7-cre; Pkd1* ^*fl/fl*^), *Tulp3* cko;*Pkd1* cko (*HoxB7-cre; Tulp3* ^*fl/fl*^ *;Pkd1* ^*fl/fl*^). cko: conditional knockout. Scale bar, 1 mm. Images adapted from (Hwang et al., 2019). **(B)** Adult-onset models. *Pax8* ^*rtTA*^ *; tetO*-cre doxycycline inducible mice were given doxycycline (Dox) starting at P28 for 2 weeks and analyzed at 18 weeks. Control (mice without *tetO*-cre), *Tulp3* cko (*Pax8* ^*rtTA*^ *; tetO-cre*; *Tulp3* ^*fl/fl*^), *Pkd1* cko (*Pax8* ^*rtTA*^ *; tetO-cre; Pkd1* ^*fl/fl*^), *Tulp3* cko;*Pkd1* cko (*Pax8* ^*rtTA*^ *; tetO-cre; Tulp3* ^*fl/fl*^ *;Pkd1* ^*fl/fl*^). Images adapted from (Legue and Liem, 2019) with permission.

Conditional knockout (cko) of *Tulp3* in renal tubules in mice using *Ksp1-Cre* or *HoxB7-Cre* caused cyst formation during development approximating ciliary loss (Hwang et al., 2019) (Figure 4A). This result indicated that a subset of TULP3-trafficked ciliary cargoes suppresses cystogenesis in normal tubules, and that lack of these molecules in the cilia underlies the disease in the *Tulp3* cko. Furthermore, *Tulp3* cko mutants showed cystic changes indistinguishable from *Tulp3*-cilia double cko mutants (Hwang et al., 2019). This result implies that TULP3/IFT-A is necessary to traffic most, if not all, crucial components of cilia-regulated cyst inhibitory signals to cilia.

Cystic kidney disease in *Tulp3* cko was slower to develop and less severe than that caused by loss of *Pkd1*. However, concomitant *Tulp3* cko had a distinctive effect on cystogenesis of *Pkd1* cko than from concomitant ciliary loss. Unlike loss of cilia, concomitant *Tulp3* cko did not inhibit cystogenesis upon PC1 loss but rather caused earlier lethality than *Pkd1* cko alone (Hwang et al., 2019), suggesting that *Tulp3* inactivation accelerated loss of renal function in the *Pkd1* cko. This genetic epistasis between *Tulp3* and *Pkd1* implied that some TULP3 ciliary cargoes suppress cystogenesis independently of polycystins during kidney development and that dysregulation of these signals may significantly contribute to the cyst growth in ADPKD.

A recent multi-group study led by the Bergmann laboratory found biallelic *TULP3* mutations in patients with progressive fibrocystic kidney disease, degenerative liver fibrosis, and hypertrophic cardiomyopathy with atypical fibrotic patterns in histopathology (Devane et al., 2022). Another multi-group study led by the Harris laboratory found monoallelic loss-of-function *IFT140* mutations in patients with mild polycystic kidney disease with limited kidney insufficiency. The authors analyzed the United Kingdom Biobank cystic kidney disease group and found probands with *IFT140* loss-of-function variants as the third most common group after *PKD1* and *PKD2* (Senum et al., 2022). Conditional knockout of another IFT-A core subunit, *Ift144*, does not suppress cystogenesis from *Pkd1* loss during embryogenesis, despite causing shortened or no cilia (Yu et al., 2022). Rather cyst number is increased arguing for a role of IFT144 cargoes in CLCI signaling (Yu et al., 2022). Although IFT140 and IFT144 are core IFT-A complex subunits (Mukhopadhyay et al., 2010; Behal et al., 2012) and regulate retrograde trafficking in cilia (Piperno et al., 1998; Iomini et al., 2001; Iomini et al., 2009), the mild polycystic kidney disease phenotype in patients with *IFT140* mutations and the lack of cyst suppression in *Pkd1; Ift144* double cko with respect to *Pkd1* cko could partly arise from pre-ciliary function of the IFT-A core in trafficking TULP3 and its cargoes.

Based on these results, we propose that a subset of TULP3/IFT-A ciliary cargoes generate **CLCI** signal(s) and can be defined experimentally by the following criteria:

(i) It is trafficked to cilia.
(ii) It could be a ciliary cargo of TULP3.
(iii) Lack causes cystic changes but milder than *Pkd1/2* cko.
(iv) Concomitant cko with *Pkd1/2* cko enhances cystic kidney phenotype during development.

## TULP3/IFT-A Ciliary Cargoes as Cilia-Localized CDCA signal(s) in Adult Kidneys

Recent epistasis data of *Tulp3* and *Pkd1* in adult mouse kidneys have provided compelling evidence for a critical role of *Tulp3* in the trafficking of the ciliary component(s) of the CDCA signal in *Pkd1* cko mice. The Liem laboratory showed that in adult mice, concomitant loss of *Tulp3* completely suppressed cystogenesis in *Pkd1* cko in adult mice at 18 weeks (Figure 4B) (Legue and Liem, 2019). *Tulp3* cko by itself at this age caused no cystogenesis (Figure 4B). However, later at 42 weeks, *Tulp3* cko did cause limited cystogenesis (Legue and Liem, 2019).

These results suggest that TULP3 traffics cilia localized cyst promoting signal(s) into cilia, which are suppressed by polycystins in normal tubules but are derepressed in adult-onset *Pkd1* cko. These signals are equivalent to the ciliary components of the CDCA. Overall, these findings provide strong evidence for a cilia-localized component(s) of the CDCA signal and delineate those as a subset of Tulp3/IFT-A ciliary cargoes in adult kidneys. To highlight their cilia-specificity and distinctiveness in action within the cilia, we term the cilia-localized component ciliary CDCA or cCDCA.

The *Pkd1* cko late-onset model offers a cyst suppressor system to test the cCDCA candidates from among the TULP3/IFT-A ciliary cargoes. The TULP3-dependent cCDCA signal(s) may be identified experimentally by the following criteria:

(i) It is trafficked to cilia.
(ii) It is a ciliary cargo of TULP3.
(iii) Lack causes no cystic changes in early adulthood.
(iv) Concomitant cko rescues adult-onset PKD in *Pkd1/2* cko at early adulthood.

The Tran laboratory found a result similar to *Tulp3* for the IFT-A peripheral subunit *Ift139/Thm1.* Concomitant loss of this IFT-A subunit suppresses cystogenesis from PC1 or PC2 loss in adult kidneys while retaining the cilia (Wang et al., 2022). TULP3 entry into cilia is not affected in *Thm1* mutants, as only the IFT-A core is required for TULP3 trafficking (Mukhopadhyay et al., 2010; Hirano et al., 2017). However, TULP3 is accumulated in cilia or in ciliary tips upon loss of IFT139 and other IFT-A core subunits, from defects in the retrograde ciliary trafficking (Mukhopadhyay et al., 2010; Hirano et al., 2017). Whether mechanisms similar to that from loss of TULP3 underlie the suppression of cystogenesis from IFT139 loss in adult-onset ADPKD is unclear. It is possible that TULP3 cargoes functioning as cCDCA signals could show abnormal accumulation in end-state ciliary levels from defects in retrograde trafficking out of cilia in the *Thm1* mutants, thereby affecting or disrupting the function of the cCDCA signals.

## Regulation of Cilia-Localized Signals in Early-vs. Late-Onset Cystogenesis by TULP3

**FIGURE 5:**
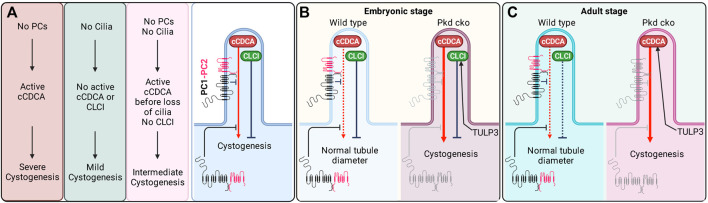
Dual roles of cilia in cystogenesis. **(A)** The genetic data (Figures 1, 4) implies **(i)** cilia-localized cilia-dependent cyst activation (**cCDCA)** signal(s) inhibited by PC1/2 in both the cell body and cilium and **(ii)** independent cilia-localized cyst inhibition (**CLCI**) signal(s). **(B)** In the embryonic stages, the cCDCA is normally weak and suppressed by polycystins. However, in the absence of polycystins, cCDCA is enhanced. TULP3 likely traffics components of the CLCI. **(C)** In the adult stage, the inhibitory arm is weaker and a lack of polycystins is sufficient to lead to strongly activated cCDCA. TULP3 likely traffics components of the cCDCA. Created in BioRender.

Recent studies of *Tulp3* mouse models have indicated complex counterregulatory cilia localized signals that positively (cCDCA) and negatively (CLCI) impact cyst growth (Figure 5A). Given the differences in dependency of these signals on polycystins for activity, the CLCI and cCDCA are likely distinct molecular entities. If TULP3/IFT-A can traffic the components for both CLCI and cCDCA signals to cilia in the absence of polycystins, why does concomitant *Tulp3* cko result in such different effects on cyst growth following early and late *Pkd1* gene inactivation?

One possibility is that TULP3/IFT-A traffics different cargoes in developing vs. adult kidneys (Figures 5B,C), perhaps to meet specific needs and tasks of the kidneys that likely differ at each stage. TULP3 inactivation would result in the ciliary exclusion of a different set of cargoes in the two stages to account for the different effects. During kidney development, TULP3/IFT-A may predominantly traffic CLCI components to cilia but few cCDCA components, which are activated following the loss of polycystins (Figure 5B). There could be redundancy between TULP3 and Tubby (or other members of the family) in trafficking cCDCA components to cilia in the developing kidney. This may allow the signal(s) to be regulated by a broader number of inputs perhaps required during development when renal tubules are forming, elongating, and branching. However, unlike in adult kidney epithelia, *Tubby* is expressed at very low levels in the developing embryonic and perinatal kidney compared to *Tulp3* (https://www.ebi.ac.uk/gxa/experiments/E-MTAB-6798/Results). In the adult kidney, TULP3/IFT-A might predominantly traffic the cCDCA component(s) unique to the adult state, but few CLCI components (until the later adult stages) (Figure 5C). The CLCI signal(s) might no longer be present or may be less effective to inhibit cyst growth at this stage when terminal tubule differentiation and maturation are complete.

Alternatively, there may be no fundamental differences in the TULP3/IFT-A regulated trafficking of cilia-signaling components between the two stages. The cCDCA may instead be trafficked by TULP3/IFT-A during development as in adult kidneys, but this regulation may be obscured by the rapid cyst growth following early inactivation of *Pkd1*. Conditional inactivation of *Tulp3* causes a stop to the ongoing ciliary trafficking of the cCDCA components. However, the already elevated cCDCA may have delayed turnover from the cilia to halt rapid cyst growth following early *Pkd1* inactivation. Residual activity of cCDCA is likely sufficient to drive considerable cyst growth that is rapidly ongoing, thus preventing a rescue of cyst growth in the *Tulp3-Pkd1* double cko during development. This scenario is consistent with the previous finding that severity of cyst growth is highly sensitive to the length of time between the initial loss of the polycystins and the subsequent involution of cilia (Ma et al., 2013). Therefore, TULP3 may regulate cCDCA in addition to the CLCI during development, but fast cyst progression in absence of Pkd1 could make such regulation difficult to detect. Genetic epistasis between TULP3 cargo and *Pkd1* mutants of varying severities will be required to unmask either cCDCA or CLCI signal during development (see next Section). In comparison, cysts grow at a much slower rate following *Pkd1* cko in adult age. The elevated level of cCDCA may drop below a threshold that is required to promote cyst growth within a time interval that is not sufficient to drive cyst growth to a significant extent.

Such counterregulatory ciliary roles are the foundational basis of the Hedgehog (Hh) pathway (Anvarian et al., 2019; Kopinke et al., 2021). We and others (Zhang et al., 2001; Huangfu et al., 2003; Norman et al., 2009; Qin et al., 2011; Mukhopadhyay et al., 2013; Somatilaka et al., 2020) have shown that cilia play an essential role by localizing both positive (e.g., Smoothened) (Corbit et al., 2005; Rohatgi et al., 2007) and negative regulators of Hh pathway, e.g., GPCR Gpr161 (Mukhopadhyay et al., 2013) and adenylyl cyclases (Somatilaka et al., 2020). They modify Gli-transcriptional factors into repressors or activators by activating or repressing cAMP-regulated protein kinase A, strictly in a cilia-dependent manner. Even the Gli transcriptional factors localize (Haycraft et al., 2005) and transit through cilia in this process (Ocbina and Anderson, 2008; Kim et al., 2009).

## Dissecting TULP3 Cargoes as Potential CLCI and cCDCA SIGNALS BY GENETIC EPISTASIS

The genetic epistasis approaches with TULP3 cargoes and *Pkd1* mutants of varying severity during kidney development or in adult kidneys would inform whether TULP3 cargoes function as likely CLCI or cCDCA signals. A decrease in the severity of *Pkd1* mutant cystic phenotype upon concomitant loss of a TULP3 cargo would argue for this cargo to function as a cCDCA signal. An increase in severity of *Pkd1* mutant cystic phenotype upon concomitant loss of a TULP3 cargo would argue for this cargo to function as a CLCI signal.

*Tulp3* deletion alone causes milder cystogenesis than *Pkd1* loss. Concomitant *Tulp3* cko in *Pkd1* cko animals did not inhibit cystogenesis but caused earlier lethality than *Pkd1* cko alone, suggesting that *Tulp3* inactivation accelerated loss of renal function in the *Pkd1* cko. Therefore, it is highly likely that TULP3 traffics cilia-localized cyst inhibition (CLCI) signal(s) during kidney development. Alternatively, Tulp3 could additionally regulate a cCDCA signal during development, but fast cyst progression in absence of *Pkd1* could make such regulation difficult to detect. Genetic epistasis between TULP3 cargo and developmental models of *Pkd1* mutants of varying severities could unmask either signal.

Lack of TULP3 in adult-onset models does not cause cystogenesis at 18 weeks. Cystogenesis in adult-onset *Pkd1-Tulp3* double cko mice is fully suppressed at this stage. Intact kidney epithelial cilia in *Tulp3* mutants argue for cilia-generated signaling rather than gross ciliary morphology defects in such suppression. Thus, it is highly likely that TULP3 traffics the ciliary component of CDCA (cCDCA) signal(s) predominantly in adult kidneys. Mild cystogenesis from *Tulp3* deletion in adult-onset models occurs much later at 42 weeks, suggesting low CLCI activity of TULP3 cargoes only at older ages. Genetic epistasis between *Tulp3* cargo mutants and *Pkd1* mutants could therefore unmask cCDCA signal(s).

The complete lack of a TULP3 cargo using a conditional knockout strategy does not equate to *Tulp3* cko that shows a selective loss of the corresponding TULP3 cargo from cilia alone without affecting the extraciliary pools. In certain cases, TULP3 cargoes that are selectively deficient in trafficking to cilia without affecting the functionality can be generated by mutating ciliary localizing signals targeted by TULP3 (e.g., for the GPCR cargo GPR161 (Hwang et al., 2021) or by targeting sequences that affect ciliary localization by TULP3 independent mechanisms (e.g., the RVxP motif for the lipidated protein ARL13B (Gigante et al., 2020), also a TULP3 cargo).

Although TULP3 loss does not affect ciliogenesis, complete lack of certain cargoes, such as ARL13B (Li et al., 2016; Seixas et al., 2016) or ARL13B-regulated INPP5E (Hakim et al., 2016), developmentally can cause ciliary disruption. These indirect effects of TULP3 cargoes on ciliary morphologies should be accounted for when performing genetic epistasis approaches between *Pkd1* mutants and TULP3 cargo cko mutants.

## Known TULP3 and IFT-A Cargoes as Potential CLCI and cCDCA Signals

Here we discuss TULP3-trafficked ciliary cargoes that might regulate both CLCI signal(s) independent of polycystins during development and cCDCA signal(s) repressed by polycystins in adult kidneys.

### ARL13B

We and others recently showed that ARL13B is a TULP3 cargo in kidney epithelia *in vivo* (Hwang et al., 2019; Legue and Liem, 2019). ARL13B is a ciliary GTPase that regulates intraciliary trafficking of lipidated cargoes (Humbert et al., 2012). Inhibiting ARL13B would block multiple lipidated cargoes bound for cilia. In further support of the TULP3-regulated CLCI, developmental deletion of *Arl13b* causes mild fibrocystic kidney disease in mice models (Li et al., 2016; Seixas et al., 2016), similar to cilia loss. The zebrafish *arl13b* (*scorpion*) allele has nephric duct dilatation phenotypes, and analysis of phenotypic rescue using *arl13b* variants in this model suggests that ciliary localization is essential for *in vivo* function of ARL13B (Sun et al., 2004; Duldulao et al., 2009).

### ARL13B Regulated Cargoes

**FIGURE 6:**
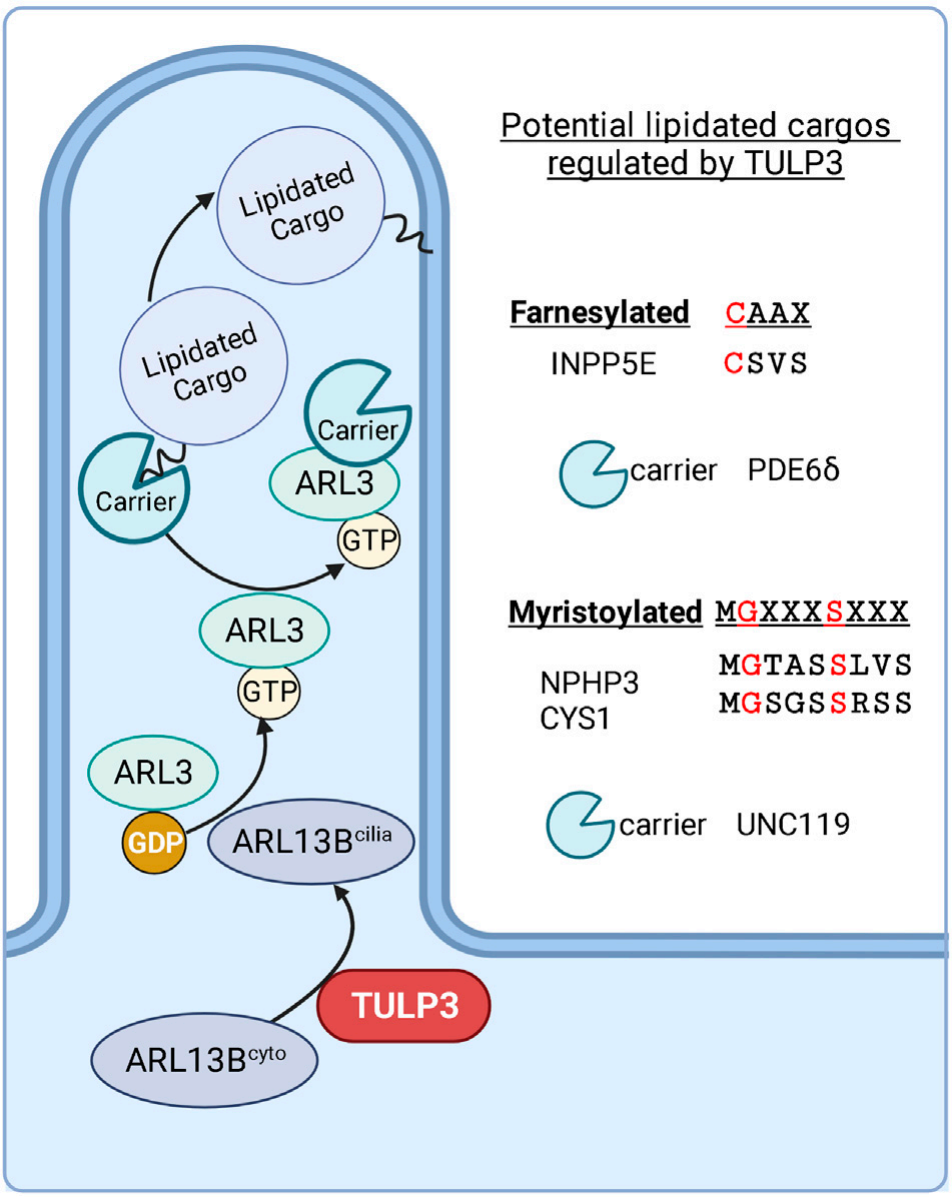
Potential lipidated cargoes of TULP3. TULP3 directs the trafficking of ARL13B to cilia. ARL13B regulates the release of farnesylated and myristoylated cargoes in the ciliary compartment *via* ARL3. Created in BioRender.

ARL13B functions as a GEF for ARL3 (Gotthardt et al., 2015; Ivanova et al., 2017). ARL3^GTP^ regulates the ciliary localization of farnesylated proteins (e.g., 5’ phosphatase INPP5E), and myristoylated proteins (e.g., NPHP3 and Cystin-1) by releasing them from their binding partners PDE6δ (Humbert et al., 2012) and UNC119B (Wright et al., 2011), respectively (Figure 6). The full list of ARL3-regulated lipidated cargoes in cilia is unknown. Residues flanking farnesylation site (CAAX box) regulate PDE6δ selectivity to cargoes (Fansa et al., 2016). The direct binding between TULP3 and certain cargoes, such as that between TULP3 and INPP5E (Humbert et al., 2012), could also factor in TULP3 mediated trafficking of these cargoes. Using IMCD3 *Tulp3* ko cell lines, we recently showed that ARL13B and INPP5E were most affected, whereas NPHP3 and Cystin-1 were comparatively less affected (Palicharla et al., 2021). We also demonstrated a significant difference in the kinetics of loss of ARL13B and ARL3-dependent lipidated proteins from the *Tulp3* cko kidney cilia: ARL13B is almost completely lost by P0, INPP5E by P5 and NPHP3 by P24. The percentage of ciliated cells and ciliary length in the collecting ducts was unchanged in *Tulp3* cko mice (Hwang et al., 2019). The farnesylated protein LKB1, which is ARL3 independent for trafficking to cilia, is not TULP3 regulated. The effectiveness for complete depletion of INPP5E in cilia in *Tulp3* cko might be related to direct binding between ARL13B and INPP5E and a requirement of such binding for effective ciliary retention of Inpp5e (Humbert et al., 2012; Qiu et al., 2021). NPHP3 is concentrated in the proximal ciliary inversin compartment by binding to NEK8 and ANKS6 that are required downstream of Inversin for NPHP3 localization (Bennett et al., 2020). Such binding might promote some retention of NPHP3 even in the absence of TULP3. Lack of *Arl3* (Schrick et al., 2006) or ARL3-dependent lipidated proteins, *Inpp5e* and *Nphp3* in developmental cko models (Wright et al., 2011; Humbert et al., 2012) or *Cystin-1* mutants (Ricker et al., 2000; Hou et al., 2002; Omori et al., 2006), causes fibrocystic disease. Nonetheless, these cilia localization experiments in *Tulp3* cko provide a potential road map for testing the most affected ARL13B regulated cargoes, such as INPP5E, as potential CLCI candidates.

### GPCRs

Multiple class A rhodopsin family GPCRs that are trafficked to cilia are TULP3 cargoes. One of these GPCRs, GPR161, is known to be ciliary in the IMCD3 cells (Mukhopadhyay et al., 2013) and expressed highly in the kidney CCD cells (Mehta et al., 2022). However, lack of GPR161 during kidney development does not cause cystogenesis (Hwang et al., 2019). Thus at least this GPCR is not a CLCI signal. Tolvaptan, an antagonist to the GPCR V2R, is the only FDA-approved drug to slow eGFR decline in patients (Torres et al., 2017; Torres et al., 2020). In addition to its prevailing basolateral plasma membrane localization, V2R was also reported to localize to the cilia (Raychowdhury et al., 2009; Sherpa et al., 2019). V2R is a Gαs coupled GPCR (Ausiello et al., 1987; Fenton et al., 2007), and Tolvaptan should prevent the cAMP level increase from Vasopressin. However, Tolvaptan treatment paradoxically increased cAMP levels in cilia in a V2R-independent manner (Sherpa et al., 2020), suggesting that such a nonspecific increase does not correspond to the role of V2R in the downstream signaling (Wang et al., 2005; Reif et al., 2011).

### Polycystins

An hypomorphic *Tulp3* mutant (*K407I*) shows cystogenesis in the embryonic kidney, but cilia are maintained (Legue and Liem, 2019). Ciliary disruption (Lin et al., 2003; Jonassen et al., 2008; San Agustin et al., 2016) or loss of ARL13B (Li et al., 2016; Seixas et al., 2016) or that of INPP5E (Hakim et al., 2016) does not cause cystogenesis during embryogenesis. Thus, the effects of the hypomorphic mutant are supposedly not only from lack of ARL13B/INPP5E trafficking to cilia. Polycystins themselves are also regulated in ciliary localization by TULP3 (Kim et al., 2014; Badgandi et al., 2017; Hwang et al., 2019), and PC2 is also partially reduced in ciliary levels in kidney epithelial cilia in the *Tulp3 K407I* mutant (Legue and Liem, 2019), which might partly explain this conundrum. PC2 trafficking to cilia is also regulated by the RVxP motif at its N-terminal end (Geng et al., 2006) while PC1 trafficking to cilia may be regulated by a similar RVxP motif at its C-terminal end (Ward et al., 2011; Su et al., 2015). The lack of the RVxP motif reduces proximity between TULP3 and PC2 (Hwang et al., 2019) but the coordination between TULP3 and the RVxP motif in the trafficking of PC2 is unclear. The *Pkd2lrm4* (E442G) mutant in the first extracellular loop is unable to localize to cilia despite an intact RVxP (Walker et al., 2019), suggesting that the RVxP motif alone is not sufficient to cause a ciliary localization (Pearring et al., 2017). TULP3’s role in regulating polycystins abundance in cilia should not affect genetic epistasis analyses between potential TULP3 cargoes and polycystins to unravel cCDCA/CLCI signals.

### Adenylyl Cyclases

Both *AC5* (Wang et al., 2018) and *AC6* (Rees et al., 2014) deletion individually reduces renal cyclic AMP and cyst growth in an orthologous mouse model of polycystic kidney disease, suggesting their role in ADPKD. A recent omics-level study of cAMP signaling components in kidney tissue showed that AC6 and AC5 are predominantly present in collecting ducts and less strong in proximal tubules (Mehta et al., 2022). AC5/6 are cilia localized. The Igarashi laboratory showed that AC5/6 are in a complex with protein A-kinase anchoring protein 150 (AKAP150) and protein kinase A (Choi et al., 2011). The authors proposed that PC2 interacts with the AC5/6 complex through its carboxy terminus. A cAMP-specific member of the phosphodiesterase family, PDE4C, is also located in renal primary cilia and interacts with the AKAP150 complex. An IFT-A core subunit IFT144 mutant (*Ift144* ^*dmhd*^) shows reduced AC3 in the neural tube cilia (Liem et al., 2012). AC3 is one of the predominant ACs in the neurulation (Somatilaka et al., 2020). Whether such a role of IFT144 in AC3 trafficking encompasses other ciliary ACs and affects kidney expressed AC5 and AC6 is currently unknown. Besides, whether such a role of IFT144 in AC trafficking involves TULP3 interactions with the core subunits of IFT-A is also unknown. Recent optogenetic and/or chemogenetic techniques for manipulating the ciliary cAMP (Hansen et al., 2020; Truong et al., 2021) could be pivotal in determining if ciliary cAMP signaling plays a role in initiating cystogenesis.

## Concluding Statement

What could be the molecular output propagated in cilia by CLCI and cCDCA signals? Based on our results showing early depletion of ARL13B and INPP5E from *Tulp3* cko kidney epithelial cilia (Hwang et al., 2019; Palicharla et al., 2021), they are strong contenders for CLCI signals. INPP5E is a 5′phosphatase that generates PI(4)P from PI(4,5)P_2_ (Kisseleva et al., 2000). If INPP5E is the CLCI signal, by generating a PI(4)P exclusive ciliary membrane domain distinct from the plasma membrane rich in PI(4,5)P_2_, it could regulate ciliary components. The CLCI signal could be activating a transcription factor in cilia that is regulatable by the ciliary microenvironment. Such an example is seen in Hh pathway where GLI2 and GLI3 transcription factors are modified in cilia upon Hh addition (Haycraft et al., 2005). Some of the Gli-similar proteins (Lichti-Kaiser et al., 2012), at least two of which, GLIS3 (Kang et al., 2009) and GLIS2 (Attanasio et al., 2007), are ciliary, and show mild cystic changes perinatally upon deletion (Kang et al., 2009) or in the sensitized background of partial *Kif3a* loss (Lu et al., 2016). The cCDCA signal could similarly be activating a transcription factor in cilia that is regulatable by ciliary cAMP. An important feature of such a transcription factor would be that it localizes to cilia (in addition to the nucleus) but its deletion would not cause adult-onset cysts. Another feature would be its regulation of cell proliferation, like GLI2 in Hh-induced proliferation of cerebellar granule cells by CyclinD1/N-Myc (Yin et al., 2020). Nonetheless, understanding how ciliary signals transduce and amplify downstream cellular effects could provide important leads to understanding cystogenesis.
